# Influence of Genetic and Non-Genetic Risk Factors for Serum Uric Acid Levels and Hyperuricemia in Mexicans

**DOI:** 10.3390/nu11061336

**Published:** 2019-06-14

**Authors:** Berenice Rivera-Paredez, Luis Macías-Kauffer, Juan Carlos Fernandez-Lopez, Marisela Villalobos-Comparán, Mayeli M. Martinez-Aguilar, Aldo de la Cruz-Montoya, Eric G. Ramírez-Salazar, Hugo Villamil-Ramírez, Manuel Quiterio, Paula Ramírez-Palacios, Sandra Romero-Hidalgo, María Teresa Villarreal-Molina, Edgar Denova-Gutiérrez, Yvonne N. Flores, Samuel Canizales-Quinteros, Jorge Salmerón, Rafael Velázquez-Cruz

**Affiliations:** 1 Centro de Investigación en Políticas, Población y Salud de la Facultad de Medicina de la Universidad Nacional Autónoma de México, Ciudad de México 04510, Mexico; bereriveraparedez7@gmail.com (B.R.-P.); jorge.salmec@gmail.com (J.S.); 2 Unidad de Genómica de Poblaciones Aplicada a la Salud, Facultad de Química, UNAM/Instituto Nacional de Medicina Genómica (INMEGEN), Ciudad de México 14610, Mexico; luisrmacias@gmail.com (L.M.-K.); hugo_villamil@hotmail.com (H.V.-R.); scanizales@inmegen.gob.mx (S.C.-Q.); 3 Consorcio Genómica Computacional, Instituto Nacional de Medicina Genómica, Ciudad de México 14610, Mexico; jfernandez@inmegen.gob.mx (J.C.F.-L.); mvillalobos@inmegen.gob.mx (M.V.-C.); sromero@inmegen.gob.mx (S.R.-H.); 4 Laboratorio de Genómica del Metabolismo Óseo, Instituto Nacional de Medicina Genómica (INMEGEN), Ciudad de México 14610, Mexico; mmaye.sol@gmail.com (M.M.M.-A.); audelacm@gmail.com (A.d.l.C.-M.); 5 Consejo Nacional de Ciencia y Tecnología (CONACYT)-Laboratorio de Genómica del Metabolismo Óseo, Instituto Nacional de Medicina Genómica (INMEGEN), Mexico City 14610, Mexico; eramirez@inmegen.gob.mx; 6 Centro de Investigación en Salud Poblacional, Instituto Nacional de Salud Pública. Cuernavaca, Morelos 62100, Mexico; mquitero@insp.mx; 7 Unidad de Investigación Epidemiológica y en Servicios de Salud, Instituto Mexicano del Seguro Social (IMSS), Cuernavaca, Morelos 62000, Mexico; paula_rzps@hotmail.com (P.R.-P.); ynflores@ucla.edu (Y.N.F.); 8 Laboratorio de Enfermedades Cardiovasculares, INMEGEN, Mexico City 14610, Mexico; mvillareal@inmegen.gob.mx; 9 Centro de Investigación en Nutrición y Salud, Instituto Nacional de Salud Pública, Cuernavaca, Morelos 62100, Mexico; edgar.denova@insp.mx; 10 UCLA Department of Health Policy and Management, Fielding School of Public Health and Jonsson Comprehensive Cancer Center, Los Angeles, CA 90001, USA

**Keywords:** Uric Acid, Hyperuricemia, Genome-Wide Association Studies, polymorphisms single nucleotide, ABCG2 gene, SLC2A9 gene, Mexican population

## Abstract

Risk of hyperuricemia is modified by genetic and environmental factors. Our aim was to identify factors associated with serum uric acid levels and hyperuricemia in Mexicans. A pilot Genome-wide association study GWAS was performed in a subgroup of participants (*n* = 411) from the Health Workers Cohort Study (HWCS). Single nucleotide polymorphisms (SNPs) associated with serum uric acid levels were validated in all the HWCS participants (*n* = 1939) and replicated in independent children (*n* = 1080) and adult (*n* = 1073) case-control studies. The meta-analysis of the whole HWCS and replication samples identified three *SLC2A9* SNPs: *rs1014290* (*p* = 2.3 × 10^−64^), *rs3775948* (*p* = 8.2 × 10^−64^) and *rs11722228* (*p* = 1.1 × 10^−17^); and an *ABCG2* missense SNP, *rs2231142* (*p* = 1.0 × 10^−18^). Among the non-genetic factors identified, the visceral adiposity index, smoking, the metabolic syndrome and its components (waist circumference, blood pressure, glucose and hyperlipidemia) were associated with increased serum uric acid levels and hyperuricemia (*p* < 0.05). Among the female HWCS participants, the odds ratio for hyperuricemia was 1.24 (95% CI, 1.01–1.53) per unit increase in soft drink consumption. As reported in other studies, our findings indicate that diet, adiposity and genetic variation contribute to the elevated prevalence of hyperuricemia in Mexico.

## 1. Introduction

Hyperuricemia is necessary for the development of gout and is associated with chronic diseases like obesity, metabolic syndrome, insulin resistance, cardiovascular disease, hypertension, chronic kidney disease, and type 2 diabetes [1,2,3,4,5]. Globally, the prevalence of hyperuricemia is estimated to be 2.0–32.1%, and it is higher in men than in women [6,7,8,9]. The prevalence of hyperuricemia in a cohort study of adults in Mexico was estimated to be 20.6% among men and 13.5% in women [9]. Additionally, the prevalence increases with age, particularly among postmenopausal women [10].

Elevated uric acid (UA) concentrations can be caused by low intestinal or renal excretion rates, and from UA overproduction that is probably due to an excess of purine precursors [11]. Epidemiological evidence suggests that the body mass index, genetic polymorphisms and purine-rich foods such as alcohol, meat, legumes and seafood, can influence serum UA concentrations [12]. Some studies have reported that serum UA concentrations are markedly heritable [13,14]. 

Genome-wide association studies (GWAS) conducted in European, American, Mexican American, Indian, Japanese and Chinese populations have identified significant associations between single nucleotide polymorphisms (SNPs) in the *SLC2A9* and *ABCG2* genes, and UA levels [15,16,17,18,19,20,21]. However, genetic epidemiological studies for UA levels are limited in the Mexican population [22]. Additionally, these studies have failed to consider hyperuricemia as a dichotomous trait, and they have not included nutritional data. The aim of this study was to identify genetic and non-genetic risk factors for hyperuricemia in Mexico. This pilot GWAS in a well characterized cohort of Mexican children and adults may provide further insight into the genetic factors affecting UA levels and their relationship to known environmental risk factors. 

## 2. Materials and Methods 

### 2.1. Study Population

We performed a pilot GWAS on the participants of the Health Workers Cohort Study (HWCS), comprised of Mexican-Mestizo subjects who were followed up between 2010 and 2012. The study design, methodology and participants’ baseline characteristics have been described in detail elsewhere [23].

The discovery sample consisted of a subgroup of 411 unrelated postmenopausal women who are HWCS participants. A first stage of validation was conducted with all the HWCS participants (*n* = 1939). Individuals <18 years of age (*n* = 85), with kidney failure (*n* = 14), missing UA concentrations (*n* = 1) and missing genotype data (*n* = 30) were excluded. After these exclusions, a total of 1936 subjects (including the 396 women from the discovery sample) comprised the study sample (Supplementary Figure S1).

The replication groups consisted of 1080 obese and normal weight children (6–12 years) and 1073 obese and normal weight adults (18–82 years) from Mexico City. These case-control studies (CCS) were designed to identify risk factors for obesity and metabolic abnormalities. The recruitment and inclusion criteria have been described elsewhere (Supplementary Figure S1) [24]. 

All study participants provided written informed consent and the ethical committees from the Instituto Mexicano del Seguro Social (No. 12CEI 09 006 14) and Instituto Nacional de Medicina Genómica approved this research project [23].

### 2.2. Measurement of Outcomes

As part of the HWCS procedures, fasting blood specimens (≥ 8 h) were obtained for each participant and serum UA levels were measured by the enzymatic colorimetric method, using the SYNCHRON CX^®^ system (Beckman Coulter CA, USA) [9,23]. The serum UA in the replication groups was determined with a commercial uricase assay (UNICEL DxC600, Beckman coulter) [24]. Hyperuricemia was defined as the UA levels ≥7.0 mg/dL in men and ≥5.8 mg/dL in women; for children it was determined using the sex and age-specific cut-off values [9,25]. 

### 2.3. Assessment of Covariates

The socio-demographic information was obtained from self-reported questionnaires [23]. The procedures for collecting biochemical assays (HDL-C, LDL-C, total cholesterol, triglycerides, glucose, and insulin levels) were followed as previously described [23,24]. The homeostasis model assessment of the insulin resistance (HOMA-IR) was determined with the formula: HOMA = (insulin μU/mL × glucose mmol/L)/22.5 [26]. Metabolic Syndrome was defined according to the International Diabetes Federation [24,27,28]. Additionally, we estimated the visceral adiposity index (VAI) using sex-specific equations [29]. The coronary heart disease (CHD) risk was calculated using a previously validated recalibration of the Framingham Coronary Heart Disease Risk Score [30]. The dietary information was obtained from a food frequency questionnaire that consists of 116 items [23].

### 2.4. Sample Genotyping and Quality Control

A pilot GWAS scan was performed in 411 unrelated postmenopausal women from the HWCS using Infinium HumanCytoSNP-12 DNA v2.1 chips, following the manufacturer’s protocol. Briefly, we genotyped 300,000 SNPs, and after stringent quality control filtering we excluded individuals with a genotyping call rate <97% from further analysis (*n* = 15). SNPs were excluded when the Minor Allele Frequency (MAF) was <0.05, the Hardy-Weinberg Equilibrium (HWE) test *p*-value was <0.0001, and the SNPs call rate was <95%. A total of 225,635 SNPs in 396 women with an overall call rate of 99.68% were used for further analysis. The study design and methodology are described in detail elsewhere [31].

Genome-wide SNP imputation was performed with the Michigan Imputation Server Web-Tool [32]. The pre-imputation quality controls were a 95% sample call rate and a Hardy-Weinberg *p* > 10^−6^ and 95% SNP call rate. After haplotype phasing with Eagle’s v2.3 algorithm [33], imputed SNPs from the Haplotype Reference Consortium (HRC) panel [34] with high genotype information content were retained for the association analysis. A total of 7.2 million SNPs were used. A linear Wald test (Quantitative) was performed for the genome-wide association analysis using EPACTS software [35]. We analyzed the UA levels using residuals and inverse normal transformation for each trait with the age, body mass index (BMI) and two principal ancestry components (PC) as covariates.

In the pediatric and adult replication groups, genotyping was performed with the expanded Multi-Ethnic Genome Array (Illumina) using the same quality control criteria. The genotypes for the selected SNPs were extracted using Plink 1.9 [24].

### 2.5. Selection of SNPs for Validation

We selected *rs3775948*, *rs1014290*, *rs11722228* (*SLC2A9*) and *rs2231142* (*ABCG2*) for validation based on the following criteria: (1) SNPs with a serum UA association with *p* < 1.0 × 10^−5^ in the discovery GWAS; (2) previously reported SNPs with an evidence of a functional effect on UA; (3) MAF ≥ 0.05; and (4) SNPs that have shown the strongest association in Mexican-American populations. We used the TaqMan assay (Applied Biosystems) to genotype the four SNPs. 

### 2.6. Statistical Analysis

The Hardy-Weinberg Equilibrium was tested in each of the study groups using the chi-square (chi2) test. Descriptive analyses of demographic and clinical variables stratified by hyperuricemia were performed in each sample. To assess the differences between groups, we conducted a chi2 test for the categorical variables and a Wilcoxon test or Student’s t-test for the continuous variables (e.g., age, BMI), respectively. To estimate the association between the serum UA and genetic *SLC2A9* or *ABCG2* variants or non-genetic factors, we performed multivariable linear regressions, adjusting for different co-variables in each model. We also used a logistic regression model to evaluate the association between hyperuricemia and non-genetic and genetic factors, adjusting for covariates. Additionally, we constructed a genetic risk score (GRS) [36] that included two SNPs: *SLC2A9 rs11722228* and *ABCG2 rs2231142*, and evaluated its association with hyperuricemia. We also analyzed interactions with diet (soft drinks, alcohol, dietary patterns, total proteins, and animal proteins) among the HWCS participants by adding an interaction term to the logistic regression models. Statistical analyses were performed using STATA software, version 14.0 (StataCorp LP, College Station, TX, USA) [37]. Linkage disequilibrium (LD) and haplotype frequencies were estimated using Haploview 4.2 [38]. The discovery and validation results were combined in a meta-analysis using an inverse variance method, assuming a random-effects model. The between-group heterogeneity was assessed with I^2^ and Cochrane’s Q with the R package meta. All *p* values presented are two-tailed, and a *p* value <0.05 was considered statistically significant. To account for multiple testing (four SNPs), a *p* value of <0.0125 was considered significant (Bonferroni-corrected *p* value).

## 3. Results

### 3.1. Characteristics of Study Participants

The anthropometric and biochemical characteristics of the HWCS subjects who participated in the discovery and validation samples are shown in Supplementary Table S1. The median age in the discovery sample was 61 years, median serum UA was 5.3 mg/dl, 46.2% were overweight and 29.8% were obese. The prevalence of metabolic syndrome was that 65.4%, and 32.8% of the participants were found to have osteopenia, while 4.2% had osteoporosis. Within the validation sample, the median age was 48 years, with a prevalence of overweightness and obesity of 42.1% and 22.8%, respectively, and a median serum UA of 5.4 mg/dl. The prevalence of hyperuricemia was lower in the validation sample compared to the discovery group (26.3% vs. 34%, respectively) (Supplementary Table S1). 

The demographic and clinical characteristics of the study groups (HWCS, adults and children) are presented in Table 1. The prevalence of hyperuricemia was similar among the different cohorts: 27.8 % among the HWCS, 22.7% in the CCS adults, and 20.2% among the CCS children, respectively. The median age, BMI, waist circumference, insulin levels, HOMA, systolic and diastolic blood pressures, serum concentrations of lipids, fasting glucose, creatinine, and uric acid levels were greater among patients with hyperuricemia, compared to the controls in the different cohorts. The prevalence of metabolic syndrome and obesity were higher among patients with hyperuricemia than in the corresponding controls. These results were similar among the different cohorts (Table 1). 

### 3.2. Non-Genetic Risk Factors and Uric Acid Levels

In all the samples, we observed that obesity, metabolic syndrome and its components (hypertriglyceridemia, hypertension, and hyperglycemia) were associated with increased uric acid levels. For example, among the HWSC participants, metabolic syndrome [males β = 0.53 (95% CI 0.31, 0.75), females β = 0.57 (95% CI 0.44, 0.71)], the visceral adiposity index [males β = 0.05 (95% CI 0.009, 0.09), females β = 0.08 (95% CI 0.06, 0.11)], BMI [males β = 0.07 (95% CI 0.05, 0.10), females β = 0.06 (95% CI 0.05, 0.08)] and smoking [males β = 0.33 (95% CI 0.04, 0.62), females β = 0.41 (95% CI 0.19, 0.63)] were positively associated with higher uric acid levels (Table 2). These results were similar with hyperuricemia and the non-genetic factors in all groups. For example, individuals in the highest VAI quartile had higher odds for hyperuricemia compared with individuals from the lowest quartile. Among the female HWCS participants, for each unit increase in the daily soft drink intake the odds ratio of hyperuricemia was 1.24 (95% CI, 1.01–1.53) (Supplementary Table S2).

Additionally, female HWCS participants with hyperuricemia had an odds ratio of 1.63 (95% CI, 1.14–2.33) for CHD compared to females without hyperuricemia, after adjusting for covariates. However, there was no association between hyperuricemia and CHD among males (Supplementary Table S3).

### 3.3. Genetic Risk Factors-Discovery Sample

**Figure 1:**
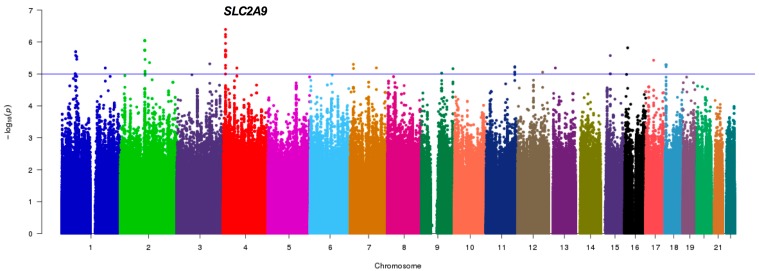
Pilot GWAS for the Serum Uric Acid (SUA) levels in the discovery sample. Manhattan plot for SUA showing the -log10 transformed *p*-value of SNPs for 411 Mexican postmenopausal women from HWCS. The blue line indicates the established threshold value of *p* < 1.0 × 10^−5^. The gene closest to the SNP with the lowest *p*-value is indicated.

Figure 1 shows the genome-wide association results in 411 postmenopausal women, a sub-cohort from the HWCS (discovery sample) with genotyped and imputed SNPs. Several SNPs showed suggestive associations with serum UA which suggests multiple loci with modest effects. None of the SNPs met the conventional criteria for a genome-wide significance. However, 47 SNPs in the solute carrier protein 2 family member 9 (*SLC2A9*) on chromosome 4 (Figure 1) met the genome-wide suggestive association threshold of *p* < 1.0 × 10^−5^.

The SNPs with a suggestive association were in a region that covered both *SLC2A9* and the adjacent gene: *WD repeat-containing protein 1 (WDR1)*. The strongest association was with the imputed intronic SNP *rs3775948* (*p* = 6.89 × 10^−7^), followed by the genotyped intronic SNP *rs1014290* (*p* = 2.62 × 10^−6^, MAF = 0.33). The linkage disequilibrium (LD) amongst them revealed that they belong to a single block within *SLC2A9* (r^2^ = 0.90) (Supplementary Figure S2). Interestingly, another associated SNP in *SLC2A9*, *rs11722228* (*p* = 9.93 × 10^−5^, MAF = 0.25), was not in LD with the lead SNP (r^2^ = 0.25). The second most significant result outside the *SLC2A9/WDR1* region was obtained with the non-synonymous SNP *rs2231142* (9.60 × 10^−5^, MAF = 0.25), in *ABCG2* (data not shown, information available upon request), also on chromosome 4.

The top suggestive associations are presented in Supplementary Table S4, with minor allele frequencies that range from 0.057 to 0.352. With the exception of the *SLC2A9* gene, there is insufficient evidence to support the role of other genes in disease pathogenesis that warrants further replication. The SNPs selected for replication were *rs3775948, rs1014290* and *rs1172222*8 of the *SLC2A9* gene, and *rs2231142* in the *ABCG2* gene.

### 3.4. Replication Analysis

In the first validation analysis, the selected SNPs were examined in the entire HWCS cohort under an additive model. The linear regression results adjusted for the main covariates indicate that four SNPs were associated with serum UA levels: *rs3775948* allele G [β = −0.39 (95% CI −0.46, −0.31), *p* = 3.1 × 10^−24^], *rs1014290* allele G [β = −0.40 (95% CI −0.48, −0.33), *p* = 1.5 × 10^−25^], *rs11722228* [β = 0.33 (95% CI 0.25, 0.41), *p* = 1.1 × 10^−15^] and *rs2231142* [β = 0.23 (95% CI 0.15, 0.31), *p* = 5.4 × 10^−8^] (Table 3). 

In the second validation, the selected SNPs also showed a significant association with the serum UA levels. Among CCS-adults, the minor alleles of *rs3775948* [β = −0.37 (95% CI −0.46, −0.27), *p* = 2.1 × 10^−16^] and *rs1014290* [β = −0.31 (95% CI −0.41, −0.22), *p* = 2.3 × 10^−10^] were negatively associated with uric acid levels, independently of other possible confounding variables. The minor alleles of *rs11722228* [β = 0.29 (95% CI 0.19, 0.39, *p* = 1.2 × 10^−8^] and *rs2231142* [β = 0.23 (95% CI 0.13, 0.32), *p* = 7.2 × 10^−6^] were associated with increased uric acid levels (Table 3).

We further analyzed the extent to which *SLC2A9* and *ABCG2* are associated with AU levels in children, and our findings were similar to the previously analyzed cohorts. The minor alleles of *rs3775948* and *rs1014290* were inversely associated with the UA levels, while the minor alleles of *rs11722228* [β = 0.42 (95% CI 0.33, 0.51), *p* = 1.6 × 10^−19^] and *rs2231142* [β = 0.24 (95% CI 0.15, 0.33), *p* = 2.3 × 10^−7^] were positively associated with increased uric acid levels (Table 3). 

To further evaluate whether *rs11722228* and *rs3775948* are independent, we analyzed the association between the serum UA and *rs3775948* conditioned by *rs11722228*. The effect of *rs3775948* in the HWCS and the replication groups decreased slightly after conditioning, but remained significant, compatible with an independent effect from these SNPs (Table 3). A meta-analysis in all samples indicated that *rs3775948*, *rs1014290*, *rs11722228* and *rs2231142* were associated with the UA levels, independently of other possible confounding variables (Table 3).

In an attempt to replicate the remaining SNPs identified in the discovery sample (Supplementary Table S4), we searched for those children and adults included in a previous study who were genotyped using the Multi-Ethnic Genotyping Array (MEGA, Illumina, San Diego, CA, USA) [24]. Of the ~13 top suggestive associations (excluding the SLC2A9 gene) that met the genome-wide suggestive association threshold of *p* < 1.0 × 10^−5^, four genotyped SNPs and five proxies were available for a replication analysis. In the replication samples (CCS-adult and CCS-children) no statistically significant associations were observed (Supplementary Table S5). 

Additionally, under the additive model, we observed statistically significant associations between hyperuricemia and the *ABCG2* SNPs and *SLC2A9* genes (Supplementary Table S6). A sensitivity analysis was performed for the association between SNPs and hyperuricemia, excluding individuals >70 years, and the results were similar; therefore we decided to maintain these individuals (data not shown). Interestingly, the odds ratio for *rs11722228* in children was slightly higher than among the HWCS participants (2.50 versus 1.71, respectively). 

**Figure 2:**
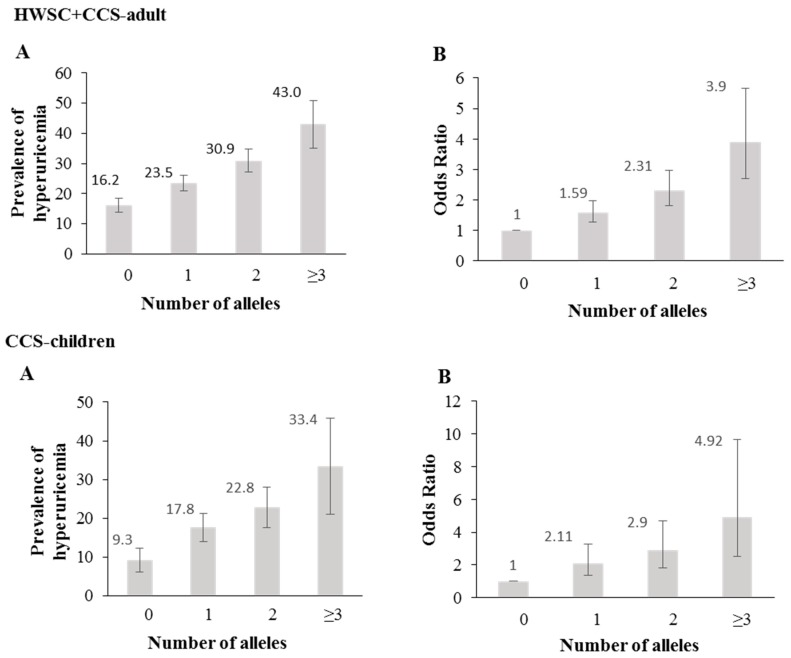
Association between a genetic risk score (GRS) and hyperuricemia. (**A**) Prevalence of hyperuricemia across the genetic risk score (rs11722228 + rs2231142) (**B**) Odds ratio of hyperuricemia for each genetic risk score (rs11722228 + rs2231142). The lines indicate 95% confidence intervals.

The genetic risk score (GRS), based on the *rs11722228* and *rs2231142* genotypes, showed that the prevalence and odds for hyperuricemia increased with the number of risk alleles (*p* < 0.0001) (Figure 2A,B). 

Among the adults from both samples and the children, the UA concentrations were modified by the *rs11722228* and *rs2231142* genotypes following an additive effect (Supplementary Figures S3–5, *p* value <0.001). However, no significant differences were observed in the glucose levels, triglycerides, BMI, and metabolic syndrome. Additionally, we did not observe statistically significant interactions between *rs11722228* and *rs2231142* regarding the previously mentioned variables. 

In the HWSC sample, there was no statistically significant association between the genetic variants of *SLC2A9* and the CHD risk, after adjusting for possible confounding variables (age, sex, uric acid levels, consumption of anti-inflammatory drugs and diuretics, creatinine concentrations and menopause) (Supplementary Table S7). We observed an association between *rs2231142* and the CHD risk under the additive model, but no co-dominant model. The results of the diet-genotype interaction analyses suggest independent effects (data not shown).

## 4. Discussion

This study examined the risk factors for hyperuricemia in a cohort with a high proportion of obesity and metabolic syndrome and in two obesity case-control studies. The prevalence of hyperuricemia in these groups was similar to other studies with metabolically compromised subjects [5,39,40,41]. A previous study of hypertensive Europeans reported a lower prevalence of hyperuricemia (17%) [5], in contrast with our results and those of another study in a European population that also observed a higher prevalence of hyperuricemia (29% and 25%, respectively) [40]. This could be due to racial or ethnic differences, age, or other factors involved in the development of hypertension. The BMI, waist circumference and body fat proportion were also associated with hyperuricemia in our study, suggesting that these factors may be contributing to the high prevalence of hyperuricemia observed in the Mexican population.

As expected, the BMI, waist circumference and body fat proportion were associated with hyperuricemia [42]. Although these are established risk factors for hyperuricemia, racial/ethnic differences have been observed in the association between the adiposity measures and UA levels [43]; therefore, their assessment in diverse populations is important. The VAI has been suggested as a better indicator for hyperuricemia risk than the BMI and waist circumference. Dong et al. observed that individuals in the highest VAI quartile had an odds ratio of 5.93 (95% CI 5.79–8.29) for hyperuricemia compared to those in the lowest quartile, although sex-specific effects were not reported [44]. The results in our study have the same direction as previous reports; however, a larger effect was observed in children and adult women from the case-control study. Additionally, the well-known association between hyperuricemia and metabolic syndrome was also observed [39,45,46,47,48] in this study, particularly among girls and adult women from the case-control study. However, it is important to note that these women are younger and have a different menopausal status, compared to the older female participants from the HWCS. Studies that have evaluated the association between hyperuricemia and metabolic syndrome by hormonal status have had conflicting results, with some reporting similar odds in both groups, [49,50] while others found a stronger relationship in premenopausal women [51]. These studies differ from ours in terms of the age range and mean BMI of the participants. Determining the effect of age or menopause on the relationship between hyperuricemia and metabolic syndrome in larger studies could help to identify high risk groups.

The Mexican population is one of the highest consumers of soft drinks, and the association between soft drink consumption and hyperuricemia is known [9,39]. A positive association between sweetened beverages and hyperuricemia, after adjusting for total calorie intake, was reported from data obtained as part of the 2004–2006 assessment of the HWCS [9]. This smaller study focused on soda intake, and the model included calorie intake, but the association with hyperuricemia was only observed among females, which could be due to the smaller sample size of males. A mechanism for this association has been proposed, according to which the phosphorylation of fructose by fructokinase, whose regulation is looser than that of hexokinase, depletes intracellular ATP, pushing UA synthesis [52]. It is likely that reducing soda consumption could be an effective public health strategy to reduce the high rates of hyperuricemia in Mexico.

Regarding genetic factors, the variants identified through a pilot GWAS proved consistent in the wider HWCS, CCS-adults and CCS-children. These were located in *SLC2A9* and *ABCG2*, two loci widely reported for their association with UA in several populations [18,19,53]. Three *SLC2A9* SNPs were selected for replication, two of these in LD. Both the LD between *rs11722228* and *rs3775948* (the third evaluated variant), as well as the conditional analysis, suggest that the effect of these SNPs on UA is independent. This extends the evidence for more than one signal in this locus [54], although the largest UA GWAS, mainly of European subjects, reports a single signal [15]. The evidence for heterogeneity in the *rs11722228* meta-analysis suggests that the effect of this SNP is larger in children. There are few serum UA genetic association studies in children; however, separate publications from the same cohort using a similar methodology are consistent, with a stronger effect of *SLC2A9* in children, at least for some SNPs [18,55]. The only UA GWAS that compares children and adults directly also found a larger genetic effect in children, although it did not reach a statistical significance [22]. Interestingly, early studies that evaluated the sex differences in the effect of *SLC2A9* on UA found evidence for interaction in some variants but not in others, although the allele frequency could account for this [56]. Our results support that allelic heterogeneity in *SLC2A9* entails heterogeneity for the age interaction. Similarly, the interaction of fructose consumption and the effect of the variants in *SLC2A9* on serum UA have been shown to differ by race/ethnicity [57]. Furthermore, inconsistent gene by diet interaction results were found in separate studies performed in Europeans and African Americans [58,59]. Dietary factors that modulated the association included legumes, red meat (women only) and vitamin C (men only) [59]. Additionally, Batt et al. observed that the C allele of *rs11942223* (*SLC2A9 gene*) was associated with lower levels of uric acid and risk of gout; however, upon exposure to the consumption of soft drinks, individuals carrying the C allele had a higher risk of gout (*p* interaction = 0.01) [60]. It is important to note that although we do not find interaction in our study, it cannot be ruled out that other genetic variants are interacting with diet. Unraveling population vs. variant specific sex and nutritional interactions in *SLC2A9* could contribute to the understanding of how this gene is regulated and how it affects the serum UA. On the other hand, sex interaction with *ABCG2’s* missense variant *rs2231142* has been reported in several populations, including Mexicans [15,22]. Allelic heterogeneity is an unlikely explanation for *ABCG2*, but it is possible that we did not observe a sex by genotype interaction with the *SLC2A9* and *ABCG2* genes because of the large proportion of postmenopausal women in this study [61].

Comparisons of the effect sizes of the association studies for the serum UA in different populations are of great interest. The present study replicated two previously reported loci of S*LC2A9* and *ABCG2* associated with serum UA levels. We compared the results of the two SNPs associated with elevated serum UA concentrations from the present study with those in Chinese, Japanese and Europeans populations. The effect sizes of these loci showed a consistent direction across the populations. For rs11722228 in *SLC2A9*, our study showed β = 0.355, Chinese β = 0.028 [62], Japanese β = 0.164 [53] and European β = 0.167 [63], and for SNP rs2231142 in *ABCG2*, our study showed β = 0.231, Chinese β = 0.046 [62], Japanese β = 0.121 [53] and European β = 0.173 [63]. It is noteworthy that the observed effect is stronger in the Mexican population.

Of the nine top suggestive associations that were available for a replication analysis, we did not observe statistically significant associations in the available replication samples. To our knowledge, these SNPs are located in genes that have not been previously associated with SUA levels or hyperuricemia, in other populations. Additional studies are required to understand the role of these genes in SUA levels in the Mexican population. Further confirmation studies are needed in other populations, to determine the main associated variants. These results could also serve as a reference and may be informative for future studies in Mexican populations.

In the HWSC sample, the glucose levels and type 2 diabetes were significantly associated with hyperuricemia in women. These results are similar to those reported by Kim et al. [45], and this could be related to our sampling strategy or to a stronger metabolic effect of UA among women, which has been previously suggested [64]. Although, the affinity of GLUT9, the gene encoded by *SLC2A9*, is mainly towards UA, it may also transport simple carbohydrates. We evaluated whether SNPs had an effect on glucose concentrations, as consistent with GWAS for glucose, but we did not observe any statistically significant associations [65,66].

No statistically significant gene-diet interactions were observed in the HWSC sample, which is likely because the dietary intake was very similar between the genotypes. Our results are similar to a previous study conducted with individuals of European descent who evaluated the interaction of the diet and *rs1014290* on UA concentrations (*p* > 0.05) [58]. However, a longitudinal study in the African-American population observed an interaction between a genetic risk score of 15 SNPs and diet on AU concentrations [59]. 

Hyperuricemia is a risk factor for cardiovascular disease [2,4,67]; however, the findings of other studies are inconsistent [22], and in our study with data from the HWSC, we only observed an association among women. A meta-analysis reported a more pronounced increased risk for CHD mortality in women [67]. On the other hand, no association was observed between the genetic variants of *SLC2A9* and the CHD risk; which may be due to the sample size within the strata and because the SNPs account for very little variability of UA. These results are similar to a previous study in the Asian population and in individuals of European ancestry [15,68]. We observed an association with *ABCG2*, but our results differ from a previous study in Mexican individuals; this could be due to variations in the study design and unmeasured confounders, so it is important to confirm these finding in future studies [22]. 

Although we observed statistically significant associations, our study has some potential limitations. First, it is a cross-sectional study that does not necessarily imply causality, although it is clear that the genetics of each individual can influence the uric acid concentrations. A second important issue is the power of the GWAS and the interaction analyses. Power is very low for both and is clearly limited to detecting associations of a greater number of genetic variants with a small effect size and/or a low minor allele frequency; this is the most likely reason we were not able to replicate the signals previously associated with hyperuricemia and serum uric acid levels in other populations. However, in the present study we identified two previously reported loci (*SLC2A9* and *ABCG2*) associated with the serum levels of UA and hyperuricemia. This suggests that our study was able to identify significant loci that are associated with serum UA levels. Third, the discovery sample only represents postmenopausal women; however we found that the prevalence of hyperuricemia in postmenopausal women was similar to the prevalence in men (34% and 30%, respectively). Fourth, in performing gene-diet interaction analyses, although diet was assessed individually and as a whole by the construction of dietary patterns, no statistically significant associations were observed because of the small sample sizes of each stratum. Additionally, the measurement error derived from the food frequency questionnaire could perhaps mask possible interactions. 

The strengths of our study include associations that were adjusted for variables accounted for in previous studies (e.g., age, sex, body fat proportion), which allow for consistency and comparability in the results. However, this did not have an effect on the estimators of this study, which is perhaps due to the fact that the variables were similarly distributed among the genotypes. The GWAS analyses in the discovery sample (22% of the total of the sample) were adjusted for ancestry to reduce the type 2 error that could be caused by the stratification of the population.

## 5. Conclusions

In conclusion, this study confirms the association between the *SLC2A9* and A*BCG2* genes and hyperuricemia. Our findings suggest the need for additional genetic studies in the Mexican population to identify genetic variants that could be used to develop genetic risk markers to identify individuals with a higher predisposition for hyperuricemia. Additional studies are needed to evaluate the gene-environment interactions associated with hyperuricemia, which could help to alleviate the burden of hyperuricemia and its complications in Mexico.

## Figures and Tables

**Table 1:** Characteristics of study groups by hyperuricemia status.

	Health Workers Cohort Study (27.8%) ***		Case-Control Study–Adults (22.7%) ***		Case-Control study–Children (20.2%) ***	
	Without Hyperuricemia *n* = 1400	With Hyperuricemia *n* = 539	Without Hyperuricemia *n* = 829	With Hyperuricemia *n* = 244	Without Hyperuricemia *n* = 862	With Hyperuricemia *n* = 218
Age (years) *	51 (39–61)	54 (42–63) ^λ^	44 (35–52)	39 (31–50) ^λ^	9 (7–10)	10 (8–11) ^λ^
Sex						
Female **	70.6 (68.3–73.0)	67.0 (63.0–71.0)	74.6 (71.7–77.6)	64.3 (58.2–70.4) ^λ^	45.2 (41.9–48.6)	44.5 (38.1–51.0)
BMI (kg/m^2^) *	26.2 (23.6–29.2)	27.9 (25.5–31.6) ^λ^	24.91 (23.14–33.8)	33.35 (30.0–36.1) ^λ^	64.5 (42.4–96.7)	96.9 (95.2–98.6) ^λ^
Overweight **	42.4 (39.8–44.9)	44.3 (0.40–48.5)	0.24 (0–0.6)	––	2.5 (1.4–3.5)	3.9 (1.4–6.5) ^λ^
Obese **	20.3 (18.1–22.3)	34.3 (30.0–38.3) ^λ^	48.5 (45.1–51.9)	76.1 (70.6–81.5) ^λ^	38.4 (35.2–41.7)	76.9 (71.4–82.3) ^λ^
Visceral adiposity index *	2.6 (1.6–3.8)	3.3 (2.3–4.7) ^λ^	2.2 (1.4–3.4)	3.3 (2.1–4.8) ^λ^	1.1 (0.6–1.9)	1.9 (1.2–3.2) ^λ^
Metabolic Syndrome **	53.6 (51.0–56.3)	75.6 (72.1–79.3) ^λ^	33.1 (29.8–36.3)	61.9 (55.7–68.0) ^λ^	13.8 (11.5–16.1)	40.8 (34.2–47.0) ^λ^
Waist circumference (cm) *	92 (85–99)	97 (90–104) ^λ^	94.0 (82.0–106.0)	104.0 (94.0–115.0) ^λ^	56.3 (31.3–86.3)	86.25 (77.8–86.3) ^λ^
Systolic blood pressure (mmHg) *	116 (106–128)	121 (111–133) ^λ^	110 (100–120)	110 (104–120) ^λ^	44 (20–71)	58.3 (33–79.7) ^λ^
Diastolic blood pressure (mmHg) *	73 (67–80)	76 (69–83) ^λ^	70 (66–80)	78 (70–80) ^λ^	65 (43–82.6)	68 (45.6–85) ^λ^
Fasting glucose (mg/dL) *	96 (90–105)	99 (93–108) ^λ^	91 (85–98)	96 (89–103.75) ^λ^	90 (85–95)	90 (86–96) ^λ^
Total cholesterol (mg/dL) *	147 (90–219)	127 (83–206) ^λ^	185 (163–211)	192 (170.2–214) ^λ^	172 (153–192)	179 (160–202) ^λ^
HDL–C(mg/dL) *	44.7 (38.0–52.7)	42.0 (36.0–49.5) ^λ^	46 (38.8–55)	40 (35–46.77) ^λ^	48 (41–56)	43 (36–50) ^λ^
Triglyceride (mg/dL) *	145 (105–195.5)	179 (135–243) ^λ^	130 (97–182.5)	171 (121.58–233.7) ^λ^	89 (63–132)	133 (91–185) ^λ^
LDL–C(mg/dL) *	118 (97–143)	126 (103–151) ^λ^	109.4 (91.2–131.08)	112.8 (94.8–130.38) ^λ^	101 (86–118.5)	108 (93–123) ^λ^
Insulin (μU/mL) ^α^	8.1 (4.3–13.3)	12.0 (6.5–18.7) ^λ^	9.9 (6.7–14.6)	13.4 (8.9–18.3) ^λ^	5.9 (3.9–9.7)	9.6 (6.5–15.9) ^λ^
HOMA ^α^*	1.9 (1.0–3.5)	3.0 (1.6–5.0) ^λ^	2.26 (1.45–3.4)	3.22 (2–4.43) ^λ^	1.33 (0.84–2.15)	2.32 (1.41–3.72) ^λ^
ALT (U/L)	21 (16–29)	25 (18–35) ^λ^	20 (15–28)	28 (19–44) ^λ^	30 (26–34)	32 (26–39) ^λ^
AST (U/L)	23 (19–29)	27 (23–34) ^λ^	21 (18–26)	24 (20–32) ^λ^	19 (16–25)	25 (19–38) ^λ^
Uric acid (mg/dL) *	4.9(4.2–5.5)	6.9(6.2–7.5) ^λ^	4.8 (4.11–5.4)	6.75 (6.1–7.5) ^λ^	4.7 (4.1–5.2)	6.5 (6.2–6.9) ^λ^

^α^ Only 1282 individuals have available insulin measurements. * Median (P25-P75). ** Percentage (95% CI). *** Prevalence of hyperuricemia. *p* values from the Kruskal-Wallis test (continuous variables) or chi2 test (categorical variables). ^λ^ *p* value <0.05. Hyperuricemia was defined as serum urate levels ≥ 7 mg/dL in males and ≥ 5.8 mg/dL in females; in children it was defined as serum urate levels ≥ 5.5 mg/dL for subjects under 7 years of age, ≥ 5.9 mg/dL for subjects aged 7–8, ≥ 6.1 mg/dL for subjects aged 9–12, ≥ 6.2 mg/dL for girls aged12 and over and ≥ 7.0 mg/dL for boys aged 12 and over.

**Table 2:** Association between metabolic syndrome, its components, diet, and smoking with serum uric acid levels.

	Health Workers Cohort Study *		Case-Control Study-Adults ***		Case-Control Study-Children ***	
	Males Beta (mg/dL, 95% CI)	Females Beta (mg/dL, 95% CI)	Males Beta (mg/dL, 95% CI)	Females Beta (mg/dL, 95% CI)	Boys Beta (mg/dL, 95% CI)	Girls Beta (mg/dL, 95% CI)
Metabolic syndrome	0.53 (0.31,0.75)	0.57 (0.44,0.71)	0.77 (0.44,1.11)	0.57 (0.43,0.73)	0.76 (0.57,0.94)	0.91 (0.71,1.1)
Metabolic syndrome components						
Waist circumference	0.60 (0.35,0.86)	0.64 (0.43,0.85)	0.99 (0.70,1.30)	0.77 (0.62–0.92)	0.93 (0.77,1.1)	1.08 (0.9,1.25)
Triglycerides (≥ 150 mg/dL)	0.54 (0.33,0.76)	0.47 (0.35,0.60)	0.77 (0.62,0.92)	0.31 (0.16,0.45)	0.29 (0.1,0.47)	0.16 (−0.03,0.35)
HDL-C ^α^	0.16 (−0.05,0.37)	0.34 (0.21,0.47)	0.38 (0.08,0.7)	0.27 (0.11,0.43)	0.09 (−0.09,0.27)	−0.02 (−0.21,0.17)
Blood pressure (≥ 130/85 mmHg, >90th percentile in children)	0.41 (0.19,0.64)	0.26 (0.11,0.41)	−0.12 (−0.53,0.27)	−0.003 (−0.24,0.23)	0.06 (−0.15,0.27)	−0.04 (−0.27,0.19)
Fasting blood glucose (≥ 100 mg/dL, ≥ 110 mg/dL in children)	−0.09 (−0.31,0.14)	0.38 (0.24,0.51)	−0.27 (−0.62,0.06)	0.37 (0.2,0.54)	1.01 (0.42,1.6)	0.94 (0.36,1.52)
Visceral adiposity index	0.05 (0.009,0.09)	0.08 (0.06,0.11)	0.08 (0.05,0.11)	0.05 (0.04,0.07)		
BMI (kg/m2) or BMI percentile	0.07(0.05,0.10)	0.06 (0.05,0.08)	0.09 (0.07,0.12)	0.06 (0.05,0.07)	0.01 (0.01,0.02)	0.02(0.01,0.02)
Soda (servings/day) **	0.04 (−0.09,0.18)	0.08 (−0.03,0.19)	--	--	--	--
Diet soda(servings/day) **	0.14 (−0.93,1.21)	0.06 (−0.05,0.18)	--	--	--	--
Smoking status						
Non-smokers	0.0	0.0				
Past smokers	0.09 (−0.15,0.33)	0.03 (−0.12,0.18)	--	--	--	--
Current smokers	0.33 (0.04,0.62)	0.41 (0.19,0.63)	--	--	--	--

^α^ Waist circumference (≥ 90 cm in males, ≥ 80 cm in females, >75th percentile in children); HDL-C (≤ 40 mg/dL in males ≤ 50 mg/dL in females and children); BMI (normal <25 kg/m^2^, overweight 25–30 kg/m^2^, obesity ≥30 kg/m^2^). * Model adjusted for age, alcohol consumption, smoking status and physical activity. ** Model: additional adjustment for energy intake. *** Model only adjusted for age.

**Table 3:** Association between SLC2A9 and ABCG2 genes with serum uric acid levels.

		Health Workers Cohort Study		Case-Control Study -Adult		Case-Control Study -Children		Meta-Analysis (All Children and Adults)		
SNP	MA	Beta (95% CI)	*p* Value	Beta (95% CI)	*p* Value	Beta (95% CI)	*p* Value	Beta (95% CI)	*p* Value	*p*-Value for Heterogeneity
rs11722228	T	0.33	1.1 × 10^−15^	0.29	1.2 × 10^−8^	0.42	1.6 × 10^−19^	0.36	1.1 × 10^−17^	0.0813
		(0.25, 0.41)		(0.19, 0.39)		(0.33, 0.51)		(0.27, 0.44)		
rs3775948	G	−0.39	3.1 × 10^−24^	−0.37	2.1 × 10^−16^	−0.43	1.1 × 10^−24^	−0.40	8.2 × 10^−64^	0.7389
		(−0.46, −0.31)		(−0.46, −0.27)		(−0.51, −0.35)		(−0.44, −0.35)		
rs1014290	G	−0.40	1.5 × 10^−25^	−0.31	2.3 × 10^−10^	−0.43	1.0 × 10^−24^	−0.40	2.3 × 10^−64^	0.6310
		(−0.48, −0.33)		(−0.41, −0.22)		(−0.51, −0.35)		(−0.44, −0.35)		
rs2231142	T	0.23	5.4 × 10^−8^	0.23	7.2 × 10^−6^	0.24	2.3 × 10^−7^	0.23	1.0 × 10^−18^	0.9426
		(0.15, 0.31)		(0.13, 0.32)		(0.15, 0.33)		(0.18, 0.28)		
rs3775948 conditioned for rs11722228	G	−0.31	1.2 × 10^−14^	−0.32	1.1 × 10^−6^	−0.32	2.9 × 10^−13^	−0.31	3.2 × 10^−35^	0.9857
		(−0.39, −0.23)		(−0.41, −0.22)		(−0.42, −0.24)		(−0.36, −0.26)		

Models included ages (<38, 38–47, 48–57, 58–67 and >67 years), sex, body mass index (normal, overweight and obesity), glucose levels (normal, intolerance and diabetes), medications (anti-inflammatories and diuretics), menopause, family cluster and creatinine levels. SUA: Serum uric acid. The meta-analysis was performed under a random effects model.

## Supplementary Materials

The following are available online at https://www.mdpi.com/2072-6643/11/6/1336/s1. Figure S1: Flowchart of study population. Table S1: Characteristics of participants in the Health Worker Cohort Study, Table S2: Association of metabolic syndrome, its components, diet and smoking with hyperuricemia, Table S3: Association between hyperuricemia and coronary heart disease from HWSC, Figure S2: LocusZoom plot of the region associated with Serum Uric Acid on chromosome 4, after imputation in the discovery sample, Table S4: Top SNPs associated with serum Uric Acid in the discovery sample, Table S5: Replication analysis of the top SNPs associated with serum uric acid levels in the Mexican population, Table S6: Multivariate logistic regression for the relationship between polymorphism in the SLC2A9 and ABCG2 genes and hyperuricemia, Figure S3: Distribution of selected biochemical traits, body mass index and metabolic syndrome in the Health Worker Cohort Study by rs11722228, rs2231142 separate and combined genotypes, Figure S4: Distribution of selected biochemical traits, body mass index and metabolic syndrome in the Adult Case Control Study by rs11722228, rs2231142 separate and combined genotypes, Figure S5: Distribution of selected biochemical traits, body mass index percentile and metabolic syndrome in the Children Case Control Study by rs11722228, rs2231142 separate and combined genotypes, Table S7: Multivariate logistic regression for the relationship between polymorphisms in the SLC2A9 gene and Coronary Heart Disease (CHD) in HWSC.
