# Supplementary material for: Influence of Genetic and Non-Genetic Risk Factors for Serum Uric Acid Levels and Hyperuricemia in Mexicans
Source: Nutrients. 2019 Jun 14;11(6):1336. doi: 10.3390/nu11061336 (PMC6627998; doi:10.3390/nu11061336)
Supplement: Supplementary file 1 [file nutrients-11-01336-s001.pdf]

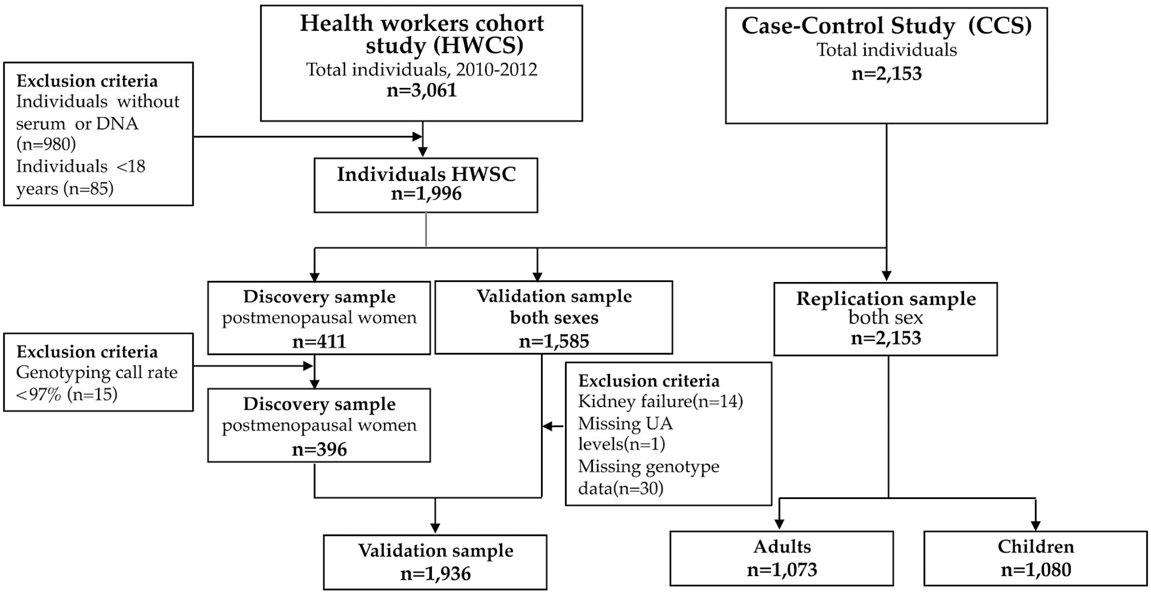

Supplementary Figure S1. Flowchart of study population

Supplementary Table S1. Characteristics of participants in the Health Worker Cohort Study

	**Discovery sample n=396**	**Validation sample n=1543**	**Total sample n=1939**
Age(years)*	61(55-68)	48(37-58)[^]	52(40-61)
Sex**			
Females**	100	61.8(59.4-64.3)[^]	69.6(67.6-71.7)
BMI (kg/m)*	27.5(25.0-30.7)	26.5(23.9-29.6)[^]	26.7(24.1-29.8)
Overweight**	46.2(41.3-51.1)	42.1(39.6-44.5)	42.9(40.7-45.1)
Obesity**	29.8(25.3-34.3)	22.8(20.7-24.8)[^]	24.2(22.2-26.1)
Body fat proportion*	45.9(41.7-49.8)	39.5(31.8-42.7)[^]	41.4(33.4-46.9)
Leisure time physical activity (METs/week)*	2.6(0.4-12.8)	5.0(0.9-18.2)[^]	12.9(3.2-30.0)
Smoking Current**	7.1(4.5-9.6)	13.8(12.0-15.5)[^]	12.9(11.4-14.5)
Past**	25.5(21.1-29.8)	27.8(25.6-30.0)	28.5(26.5-30.5)
Metabolic Syndrome**	70.2(65.6-74.7)	57.0(54.6-60.0)[^]	59.8(57.6-62.0)
Waist circumference (cm)*	95(88-103)	93(86-100)[^]	94(86-101)
Systolic blood pressure (mmHg)*	123(138-112)	117(107-127)[^]	118(108-129)
Diastolic blood pressure (mmHg)*	72(67-79)	75(68-81)[^]	74(68-81)
Fasting glucose(mg/dL)*	98(92-106)	97(90-105)	97(90-105)
HDL-C(mg/dL)*	45(38-54)	44(37-51)[^]	44(37-52)
Triglyceride (mg/dL)*	161(120-211)	154(108-207)	156(112-209)
Total cholesterol (mg/dL)*	123.4(83.3-178.3)	147.5(87.8-222.8)[^]	141.9(86.2-214.4)
LDL-C(mg/dL)*	133(160-105)	117(97-142)[^]	120(98-146)
Insulin (μU/ml)[α]*	8.8(5.1-13.1)	9.0(4.6-15.5)	8.9(4.7-15.2)
HOMA[α]	2.2(1.2-3.5)	2.2(1.1-4.1)	2.2(1.1-4.0)
ALT (U/L)*	20(16-27)	22(16-32)[^]	22(16-31)
AST (U/L)*	24(21-30)	24(20-30)	24(20-30)
Uric acid (mg/dL)*	5.3(4.4-6.1)	5.4(4.5-6.4)[^]	5.3(4.5-6.4)
Hyperuricemia**	33.8(29.2-38.5)	26.3(24.0-28.4)[^]	27.8(25.8-29.7)
Femoral neck Bone Mineral Density (g/cm2)*	0.88(0.80-0.97)	0.98(0.88-1.08)[^]	0.96(0.86-1.06)
Osteoporosis**	4.3(2.3-6.3)	1.7(1.0-2.4)[^]	2.2(1.6-2.9)
Osteopenia**	32.8(28.2-	19.1(17.1-21.1)[^]	22.1(20.0-24.0)
Spine BMD (g/cm^2)*	1.00(0.90-1.09)	1.12(1.01-1.22)[^]	1.09(0.98-1.20)
Total energy intake (kcal/day)*	1688(1244-2129)	1792(1353-2377)[^]	1761(1329-2335)
Carbohydrate intake (% energy)*	67.5(61.3-72.6)	65.4(59.4-71.0)[^]	65.9(59.7-71.4)
Protein intake(% energy)*	12.5(10.9-14.7)	12.4(10.8-14.1)	12.4(10.8-14.2)
Polyunsaturated fat intake (% energy)*	4.1(3.3-4.9)	4.2(3.5-5.0)[^]	4.2(3.5-5.0)
Saturated fat intake (% energy)*	6.9(5.7-8.7)	7.1(5.6-8.9)	7.1(5.6-8.8)
Fiber intake (mg/day)*	24.8(18.6-34.6)	24.8(17.9-33.2)	24.8(18.0-33.4)
Alcohol(g/day)*	0.3(0.0-1.4)	1.01(0.2-4.0)[^]	0.8(0.04-3.2)
Soda (servings/day)***	0.29(0.47)	0.51(0.75)	0.46(0.70)
Diet soda(servings/day) ***	0.06(0.27)	0.07(0.48)	0.07(0.45)

*Median (P25-P75). **Percentage (95% CI). ***Mean (SD). P values from Wilcoxon test or Student's t-test (continuous variables) or chi2 test (categorical variables). [α]Only 1282 individuals have available insulin measurements. [^]P value <0.05

Supplementary Table S2. Association between metabolic syndrome, its components, diet, smoking and hyperuricemia

	HWCS*		CCS-Adults***		CCS-Children***	
	Males	Females	Males	Females	Boys	Girls
	OR (95% CI)	OR (95% CI)	OR (95% CI)	OR (95% CI)	OR (95% CI)	OR(95% CI)
Metabolic syndrome (MS)	2.53 (1.69-3.77)	2.57 (1.91-3.46)	2.12 (1.24-3.62)	4.24 (2.88-6.24)	2.58 (1.59-4.16)	6.44 (3.77-11.01)
MS components						
Waist circumference α	1.01 (0.99-1.03)	0.99 (0.97-1.01)	2.47 (1.40-4.37)	4.13 (1.96-8.71)	2.59 (1.67-4.05)	5.66 (3.19-10.1)
Triglycerides (≥ 150 mg/dL)	2.49 (1.68-3.70)	2.10 (1.62-2.72)	1.74 (1.04-2.93)	2.67 (1.86-3.84)	2.01 (1.27-3.15)	3.45 (2.15-5.56)
HDL-C α	1.83 (1.27-2.66)	1.35 (1.03-1.76)	2.03 (1.21-3.41)	2.27 (1.48-3.49)	1.52 (0.96-2.39)	2.25 (1.37-3.69)
Blood pressure (≥ 130/85 mmHg, >90th percentile in children)	1.59 (1.08-2.32)	1.58 (1.19-2.10)	1.76 (0.88-3.52)	1.37 (0.76-2.54)	--	--
Fasting blood glucose (≥ 100 mg/dL , ≥ 110 mg/dL in children)	1.02 (0.70-1.47)	1.75 (1.35-2.27)	1.45 (0.82-2.57)	3.31 (2.25-4.86)	1.39 (0.80-2.41)	1.12 (0.51-2.46)
Visceral adiposity index						
1st Quartile	1.0	1.0	1.0	1.0	1.0	1.0
2nd Quartile	1.96 (1.02-3.78)	1.59 (1.00-2.52)	1.18 (0.46-3.00)	2.89 (1.23-6.81)	1.41 (0.60-3.34)	0.80 (0.30-2.13)
3rd Quartile	1.84 (0.89-3.82)	2.00 (1.17-3.44)	1.43 (0.47-4.38)	5.04 (1.96-12.93)	3.15 (1.06-9.34)	2.68 (1.0-7.20)
4th Quartile	3.12 (1.17-8.32)	2.15 (1.01-4.56)	1.68 (0.36-7.96)	6.24 (1.89-20.55)	7.16 (1.48-34.53)	7.52 (1.97-28.72)
BMI (kg/m2), Normal α	1.0	1.0	1.0	1.0	1.0	1.0
Overweight α	2.33 (1.48-3.69)	1.45 (1.05-2.00)	--	--	--	--
Obesity α	3.06 (1.78-5.34)	2.64 (1.88-3.70)	3.75 (2.02-6.98)	3.64 (2.25-5.86)	4.21 (2.17-8.17)	13.43 (4.98-36.19)
Soda (servings/day)**	1.04 (0.83-1.30)	1.26 (1.02-1.56)	--	--	--	--
Diet soda(servings/day) **	1.20 (0.21-6.95)	0.94 (0.68-1.31)	--	--	--	--
Smoking status, Nonsmokers	1.0	1.0				
Past smokers	0.77 (0.50-1.19)	0.98 (0.72-1.35)	--	--	--	--
Current smokers	1.15 (0.33-1.87)	2.35 (1.53-3.62)	--	--	--	--

Hyperuricemia was defined as a serum urate levels ≥ 7mg/dL in males and ≥ 5.8 mg/dL in females; in children was defined as a serum urate levels ≥ 5.5 mg/dL for subjects under 7 years of age, ≥ 5.9 mg/dL for subjects age 7-8, ≥ 6.1 mg/dL for subjects age 9-12, ≥ 6.2 mg/dL for girls 12 and over and ≥ 7.0 mg/dL for boys 12 and over. α Waist circumference (≥ 90 cm in males ≥ 80 cm in females); HDL-C (≤ 40 mg/dL in males ≤ 50 mg/dL in females); BMI (normal <25 kg/m^2, overweight 25-<30kg/m^2, obesity ≥30 kg/m^2) *Model adjusted for age, alcohol consumption, smoking status and physical activity. ** Model: additional adjustment for energy intake. ***Model only adjusted for age.

Supplementary Table S3. Association between hyperuricemia and Coronary heart disease from Health Workers Cohort Study

HWSC	Acid uric	Participants without CHD n(%)	Participants with CHD n(%)	Crude OR (95% CI)	P value	Model I OR (95% CI)	P value	Model II OR (95% CI)	P value
Total	Normal	971(75.6)	314(68.7)	1.0		1.0		1.0	
	Hyperuricemia	330(25.4)	143(31.3)	1.34(1.06-1.69)	0.014	1.18(0.91-1.54)	0.208	1.36(1.00-1.85)	0.047
Males	Normal	274(68.5)	114(71.7)	1.0		1.0		1.0	
	Hyperuricemia	126(31.5)	45(28.3)	0.86(0.57-1.29)	0.459	1.06(0.61-1.81)	0.842	0.76(0.40-1.42)	0.384
Females	Normal	697(77.4)	200(67.1)	1.0		1.0		1.0	
	Hyperuricemia	204(22.6)	98(32.9)	1.67(1.26-2.23)	0.0004	1.33(0.96-1.82)	0.070	1.63(1.14-2.33)	0.008

SUA: Serum uric acid; Hyperuricemia: 7.0 mg/dL for males, and 5.8mg/dL for females. Model I: Adjustment for age (<38, 38-47, 48-57, 58-67 and >67 years) and sex. Model II: Additional adjustment for medications (anti-inflammatories and aspirin), creatinine levels, alcohol intake (quintile), leisure time physical activity (quintile), smoking and menopause. ** Exclusion of individuals with previous CHD.

Supplementary Figure S2. LocusZoom plot of the region associated with Serum Uric Acid on chromosome 4, after imputation in the discovery sample. SNPs are color-coded according to the linkage disequilibrium (LD) with the genotyped SNP (purple diamond) indicated by the arrow.

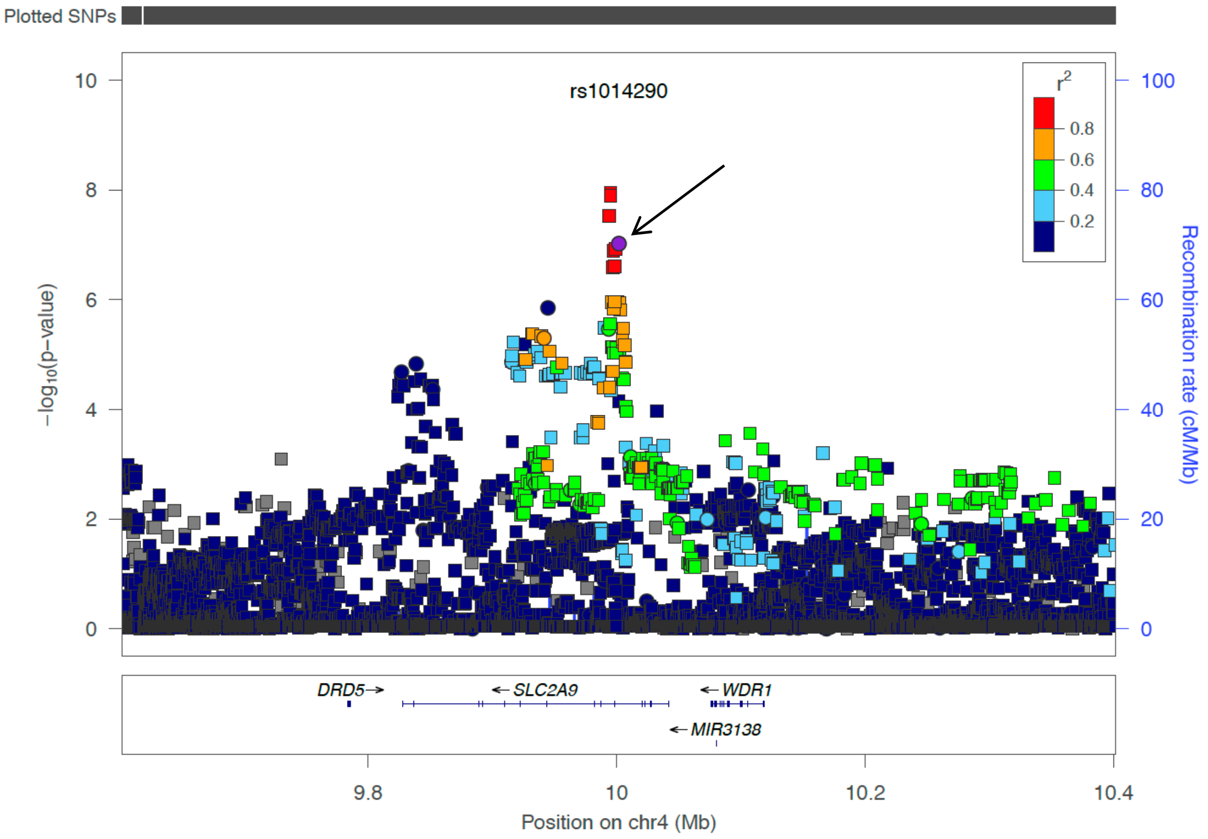

Supplementary Table S4. Top SNPs associated with serum Uric Acid in the discovery sample.

SNP ID	Chr	Position[a]	*Gene*	A1[b]	A2	MAF[c]	β	SE	*P-value*
rs338229	1	58942533	*DAB1|OMA1*	C	T	0.19	0.530	0.109	2.01×10^{-6}
rs1418978	1	56971724	*PPAP2B*	C	T	0.32	-0.419	0.094	9.81×10^{-6}
rs7606088	2	105515963	*near to MRPS9*	C	G	0.337	0.446	0.089	9.06×10^{-7}
rs11124085	2	106758629	*UXS1*	G	A	0.12	0.596	0.127	3.51×10^{-6}
rs2139266	3	140526480	*TRIM42-SLC25A36*	*C*	T	0.352	0.391	0.084	4.84×10^{-6}
rs3775948	4	9995256	*SLC2A9*	G	A	0.344	0.447	0.447	4.05×10^{-7}
rs1014290	4	10001861	*SLC2A9*	G	A	0.332	0.419	0.087	2.62×10^{-6}
rs6597576	9	135388415	*C9orf171*	A	T	0.096	-0.640	0.140	6.87×10^{-6}
rs4936823	11	123395713	*GRAMD1B*	G	T	0.321	0.401	0.087	5.93×10^{-6}
rs117226408	12	107704081	*near to BTBD11*	A	G	0.057	0.815	0.180	8.81×10^{-6}
rs1326384	13	28356322	*GSX1*	C	T	0.137	0.38	0.083	6.51×10^{-6}
rs76579941	15	41144467	*SPINT1*	G	A	0.080	-0.747	0.156	2.66×10^{-6}
rs3759797	15	41144467	*RP11-532F12.5*	A	C	0.069	-0.747	0.167	9.92×10^{-6}
rs36014496	16	12930516	*CPPED1-SHISA9*	A	T	0.069	0.812	0.166	1.52×10^{-6}
rs138486365	18	5860062	*near to TMEM200C*	A	G	0.277	-0.432	0.093	5.17×10^{-6}

[a]Chromosomal position (Mb) based on human genome build 19, dbSNP build 37.

[b]Allele minor.

[c]Minor allele frequency (MAF) estimated from the discovery samples.

Supplementary Table S5. Replication analysis of the top SNPs associated with serum uric acid levels in the Mexican population.

		**HWCS***		**CCS-adult**		**CCS-children**	
SNP	MA	Beta (95% CI)	P value	Beta (95% CI)	P value	Beta (95% CI)	P value
rs1418978**	C	-0.42 (-0.60, 0.24)	9.81 x 10^{-6}	0.07 (-0.02, 0.167)	0.122	0.06 (-0.02, 0.152)	0.140
**rs338229**	**C**	**0.53 (0.32, 0.74)**	**2.01 x 10^{-6}**	**0.03 (-0.08, 0.141)**	**0.583**	**0.01 (-0.09, 0.12)**	**0.791**
rs7606088**	C	0.45 (0.27, 0.62)	9.06 x 10^{-7}	-0.13 (-0.23, -0.03)	0.009	0.03 (-0.06, 0.12)	0.568
**rs11124085**	**G**	**0.60 (0.35, 0.85)**	**3.51 x 10^{-6}**	**0.02 (-0.13-0.16)**	**0.832**	**0.02 (-0.12, 0.15)**	**0.790**
rs2139266	T	0.39 (0.23, 0.56)	4.84 x 10^{-6}	-0.05 (-0.14, 0.05)	0.346	-0.009 (-0.10, 0.08)	0.840
**rs6597576****	**A**	**-0.64 (-0.91,-0.37)**	**6.87 x 10^{-6}**	**0.03 (-0.14, 0.2)**	**0.720**	**-0.08 (-0.24, 0.09)**	**0.353**
rs1326384	C	0.38 (0.22-0.54)	6.51 x 10^{-6}	0.04 (-0.04, 0.13)	0.318	-0.06 (-0.14, 0.03)	0.181
rs36014496**	A	0.81 (0.49, 1.14)	1.52 x 10^{-6}	-0.15 (-0.28, -0.009)	0.036	-0.06 (-0.19, 0.06)	0.324
**rs138486365****	**A**	**-0.43 (-0.61, -0.25)**	**5.17 x 10^{-6}**	**-0.05 (-0.15, 0.04)**	**0.275**	**0.009 (-0.08, -0.097)**	**0.849**

*The HWCS (n=411). ** Proxies for rs1418978, rs7606088, rs6597576, rs36014496 and rs138486365; were rs4912314, rs6719286, rs12005531, rs12929323 and rs3936100, respectively. Models included age (<38, 38-47, 48-57, 58-67 and >67 years), sex, body mass index (normal, overweight and obesity), glucose levels (normal, intolerance and diabetes), medications (anti-inflammatories and diuretics), menopause, family cluster and creatinine levels.

Supplementary Table S6. Multivariate logistic regression for the relationship between polymorphisms in the *SLC2A9* and *ABCG2* genes and hyperuricemia

		**HWCS**		**CCS-adult**		**CCS-children**		**Meta-analysis (all children and adults)**		
SNP	MA	OR (95% CI)	P value	OR (95% CI)	P value	OR (95% CI)	P value	OR (95% CI)	P value	P-value for heterogeneity
rs11722228	T	1.71 (1.44-2.0)	3.5×10^{-10}	1.38 (1.08-1.75)	0.009	2.50 (1.93-3.27)	8.8×10^{-12}	1.80 (1.26-2.57)	3.6×10^{-8}	<0.01
rs3775948	G	0.59 (0.50-0.69)	2.7×10^{-10}	0.59 (0.46-0.75)	1.3×10^{-5}	0.45 (0.35-0.58)	1.5×10^{-9}	0.56 (0.48-0.64)	1.2×10^{-34}	0.17
rs1014290	G	0.60 (0.51-0.71)	2.9×10^{-9}	0.57 (0.45-0.72)	4.5×10^{-6}	0.46 (0.35-0.59)	2.1×10^{-9}	0.56 (0.48-0.65)	2.5×10^{-37}	0.15
rs2231142	T	1.38 (1.17-1.63)	1.8×10^{-4}	1.46 (1.15-1.87)	0.002	1.42 (1.11-1.83)	0.006	1.42 (1.24-1.63)	1.8×10^{-96}	0.93
rs3775948 conditioned for rs11722228	G	0.64 (0.52-0.79)	4.0×10^{-5}	0.61 (0.47 – 0.79)	0.0002	0.56 (0.42 – 0.73)	3.8×10^{-5}	0.61 (0.53 – 0.70)	6.4×10^{-12}	0.72

Models included age (<38, 38-47, 48-57, 58-67 and >67 years), sex, body mass index (normal, overweight and obesity), glucose levels (normal, intolerance and diabetes), medications (anti-inflammatories and diuretics), menopause, family cluster and creatinine levels. SUA: Serum uric acid. Hyperuricemia: ≥ 7.0 mg/dL for males, and ≥ 5.8mg/dL for females; in children was defined as a serum urate levels ≥ 5.5 mg/dL for subjects under 7 years of age, ≥ 5.9 mg/dL for subjects age 7-8, ≥ 6.1 mg/dL for subjects age 9-12, ≥ 6.2 mg/dL for girls 12 and over and ≥ 7.0 mg/dL for boys 12 and over.

Supplementary Figure S3. Distribution of selected biochemical traits, body mass index and metabolic syndrome in the Health Worker Cohort Study by rs11722228, rs2231142 separate and combined genotypes.

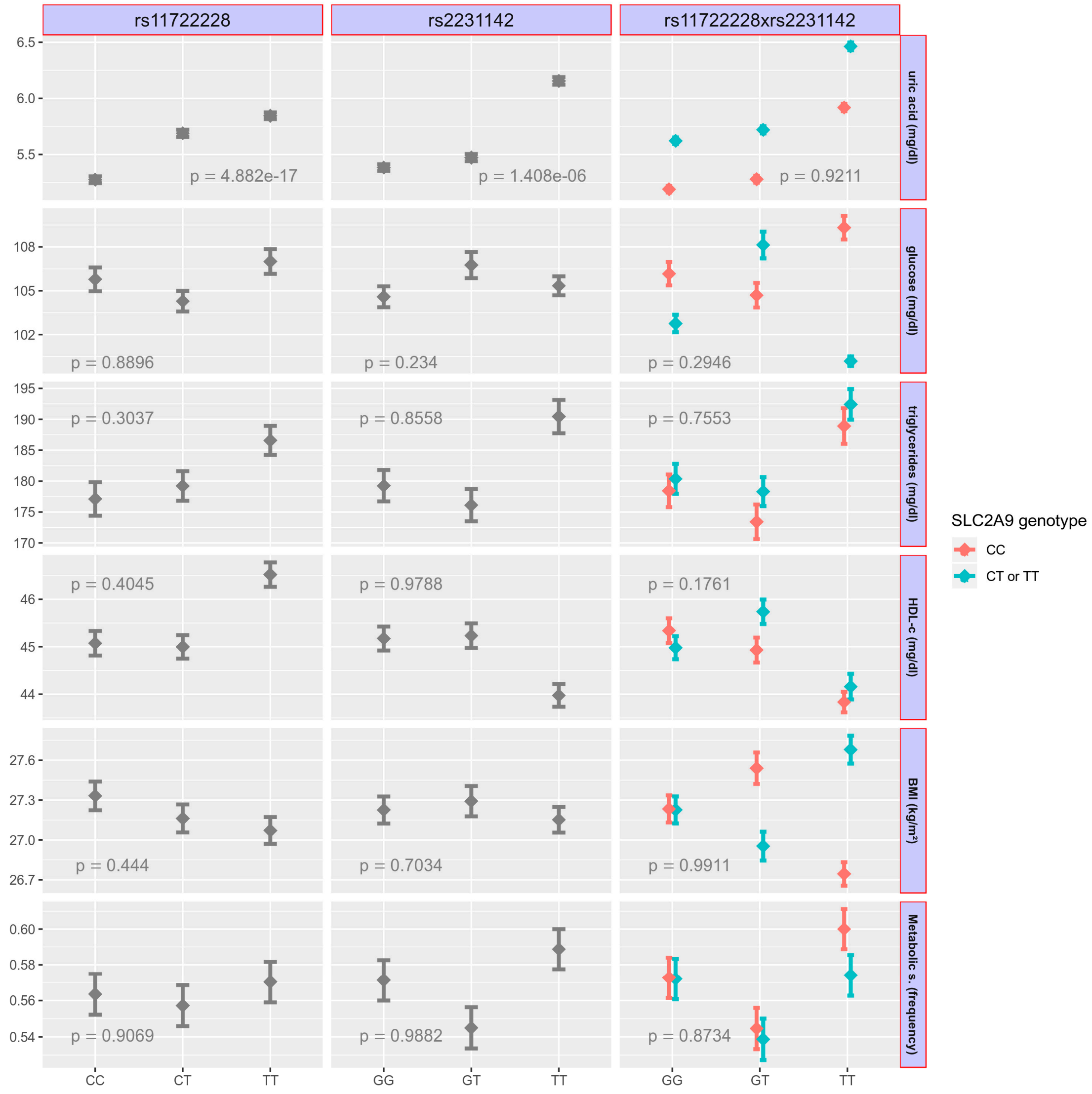

Supplementary Figure S4. Distribution of selected biochemical traits, body mass index and metabolic syndrome in the Adult Case Control Study by rs11722228, rs2231142 separate and combined genotypes.

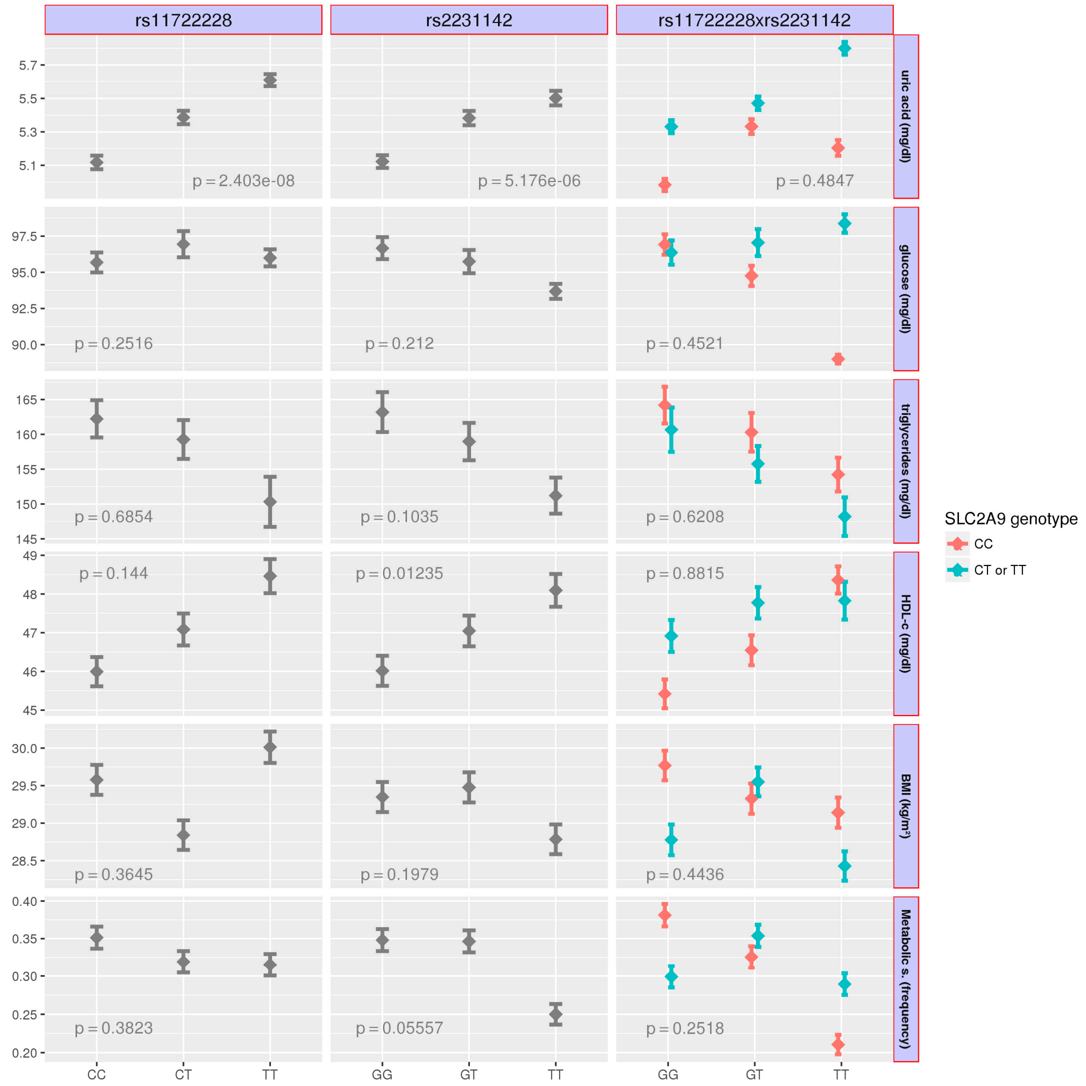

Supplementary Figure S5. Distribution of selected biochemical traits, body mass index percentile and metabolic syndrome in the Children Case Control Study by rs11722228, rs2231142 separate and combined genotypes.

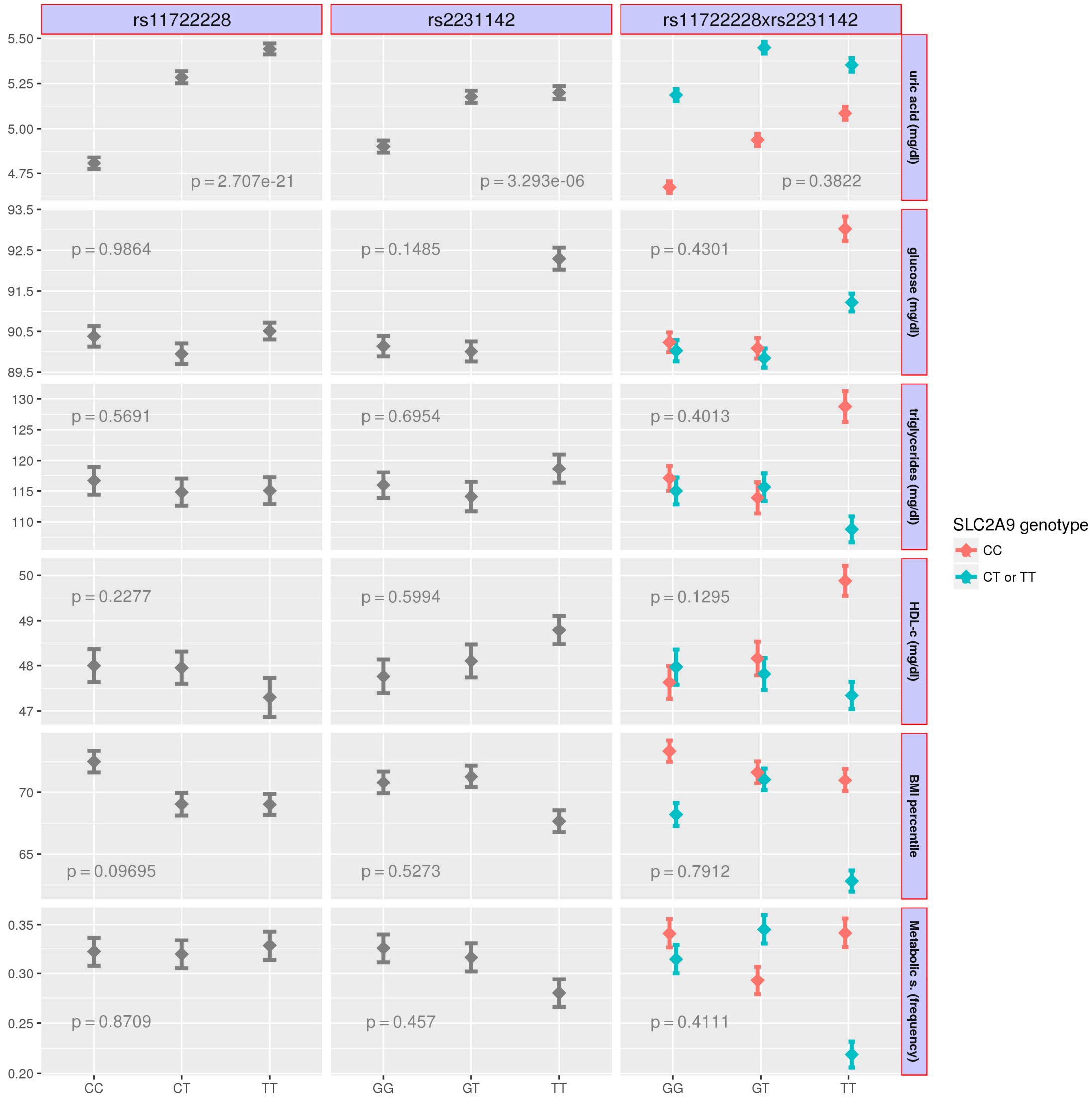

Supplementary Table S7. Multivariate logistic regression for the relationship between polymorphisms in the SLC2A9 gene and Coronary Heart Disease (CHD) in Health Workers Cohort Study

SNP	Allele	Crude OR(95% CI)	P value	Model I OR(95% CI)	P value	Model II OR(95% CI)	P value
**rs1014290**	G	0.92(0.79-1.08)	0.328	0.94(0.78-1.12)	0.486	0.97(0.79-1.20)	0.785
**rs3775948**	G	0.97(0.83-1.13)	0.682	0.99(0.83-1.19)	0.944	1.04(0.85-1.28)	0.697
**rs11722228**	T	1.01(0.85-1.20)	0.903	1.06(0.87-1.29)	0.552	1.10(0.88-1.38)	0.406
**rs2231142**	T	0.76(0.63-0.91)	0.002	0.76(0.63-0.93)	0.008	0.78(0.62-0.98)	0.030

Model I: Adjustment for (<38, 38-47, 48-57, 58-67 and >67 years) and sex. Model II: Additional adjustment for medications (anti-inflammatories and aspirin), creatinine levels, alcohol intake (quintile), leisure time physical activity (quintile), menopause, family cluster and smoking. ** Exclusion of individuals with previous CHD.
